# Effect of Aluminosilicates’ Particle Size Distribution on the Microstructural and Mechanical Properties of Metakaolinite-Based Geopolymers

**DOI:** 10.3390/ma16145008

**Published:** 2023-07-14

**Authors:** Jan Kohout, Petr Koutník, Pavlína Hájková, Eliška Kohoutová, Aleš Soukup, Mohammadtaghi Vakili

**Affiliations:** 1 ORLEN UniCRE a.s., Revoluční 1521/84, 400 01 Ústí nad Labem, Czech Republic; petr.koutnik@orlenunicre.cz (P.K.); pavlina.hajkova@orlenunicre.cz (P.H.); eliska.kohoutova@orlenunicre.cz (E.K.); ales.soukup@orlenunicre.cz (A.S.); mvakili1981@yahoo.com (M.V.); 2 Department of Material Science, Faculty of Mechanical Engineering, Technical University of Liberec, Studentská 1402/2, 461 17 Liberec, Czech Republic

**Keywords:** metakaolinite, particle size, geopolymer, metakaolin, claystone, characteristics, mechanical properties

## Abstract

The present study focused on investigating the differences in properties between calcined and milled aluminosilicates with different particle size distributions. Two types of clay, i.e., kaolin and kaolinitic claystone, were subjected to calcination at 750 °C, and subsequent milling to obtain different fractions with distinct particle size distributions. These fractions were then combined with a potassium alkaline activator and quartz sand in a 50:50 weight ratio to form a geopolymer composite. The geopolymer binders were then characterized using a mercury intrusion porosimeter (MIP), scanning electron microscopy (SEM), and a rotary rheometer. Mechanical tests were conducted on the geopolymer composites prepared from aluminosilicates with varying particle size distributions. The findings indicated that aluminosilicates with a finer particle size distribution exhibited higher levels of dissolved aluminum (10,000 mg/kg) compared to samples with coarser particle size distributions (1000 mg/kg). Additionally, as the particle size distribution decreased, the dynamic viscosity of the geopolymer binders increased, while the average pore size decreased. Finally, the mechanical properties of the geopolymer composites derived from both tested aluminosilicates demonstrated a decline in performance as the mean particle size increased beyond 10 µm.

## 1. Introduction

Over the past five decades, there has been extensive research on geopolymers, which are inorganic materials with a three-dimensional network structure [1,2,3]. Geopolymers are increasingly being considered as a viable alternative to ordinary Portland cement due to their remarkable mechanical properties, short setting time, high resistance to high temperatures and chemicals (including organic solvents and acids), as well as their low carbon emissions [4,5,6]. These materials have found wide-ranging applications in various industrial practices such as building materials [7,8], decorative and restoration materials [9], immobilizers of toxic waste [10,11], catalysts [12,13], coatings [14,15], materials for 3D printing [16,17], and fiber-reinforced geopolymer composites [18,19,20].

Geopolymers are produced by combining aluminosilicate source materials with an alkaline activator. This process involves partially dissolving powdered aluminosilicates in a liquid alkaline activator at room temperature, followed by polycondensation reactions that form a three-dimensional polymer network, resulting in the hardening of the geopolymer binder [21,22,23]. The most commonly used alkaline activators are alkali silicates (water glass), or their mixtures and aqueous alkali metal hydroxides [24,25,26]. Industrial by-products such as fly ashes [27,28], volcanic ashes [29,30], blast furnace slag [31,32], demolition wastes [33], rice husk ashes [34,35], and calcined kaolinite-rich rocks such as metakaolins are frequently employed as aluminosilicates for geopolymer synthesis [26,36,37].

The properties of geopolymers are significantly influenced by several factors, including the selected aluminosilicate [21,38,39,40,41], the type of alkali cation (sodium or potassium) [42], the Si/Al ratio [43,44], K(Na)/Si [45], or K(Na)/Al [36] molar ratio, water content [8,46,47], and the curing condition [48,49]. Additionally, the properties of the solid aluminosilicate component, e.g., particle size distribution, play a crucial role in the geopolymerization process, particularly in terms of the aluminosilicate’s ability to dissolve in the alkaline activator. Any undissolved solid aluminosilicate directly affects the physical properties of the resulting geopolymer, as it becomes part of the final product [35,47,49,50]. Particle size also has a significant impact on the viscosity, setting time, and mechanical strength of geopolymers [27,51].

Despite the importance of particle size distribution in aluminosilicate materials, only a few studies have investigated its effect on the properties of geopolymers. For instance, Li et al. [52] studied the particle size’s influence in coal gangue-based geopolymers and identified an optimal particle size of 200 mesh. In another study, the effect of red mud particle size fractions on the properties of geopolymers was examined by Zhang et al. [51]. The obtained results revealed that fluidity decreased initially and then increased with decreasing particle fraction. Assi et al. [27] compared four commercial fly ashes with different particle size distributions and found that finer particle size distributions led to enhanced compressive strength. Other authors, such as Xiong et al. [28] and Sevim and Demir [53], have also investigated the effect of fly ash particle size distribution on geopolymer properties. Gonçalves et al. [23], on the other hand, tested two commercial metakaolins with different particle sizes and amorphities and found that the particle size had no effect on the mechanical strength.

The aim of present research is to investigate how various particle size distributions of aluminosilicates affect the mechanical and microstructural characteristics of geopolymer binders and composites made from metakaolinite. To achieve this, calcined kaolin or calcined kaolinitic claystone were produced using identical conditions, and the resulting calcinates were then ground to different sizes. These different grain sizes were used as the raw aluminosilicates in the creation of the geopolymer binders. In total, six fractions with varying particle size distributions were investigated for both types of aluminosilicates.

## 2. Materials and Methods

### 2.1. Materials

**Figure 1:**
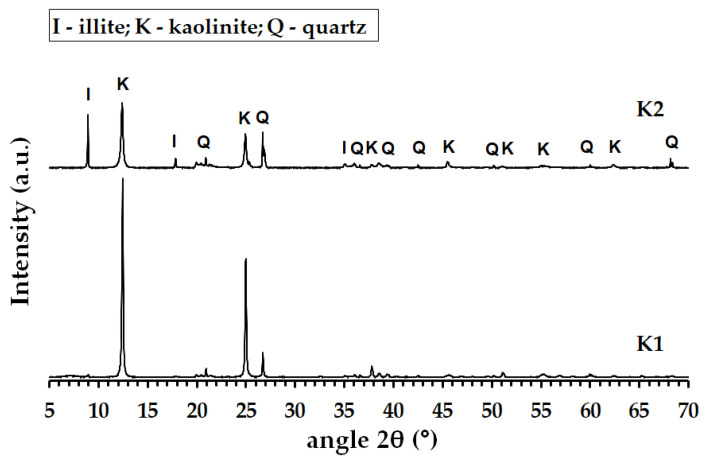
The X-ray diffraction (XRD) pattern of the K1 and K2.

**Figure 2:**
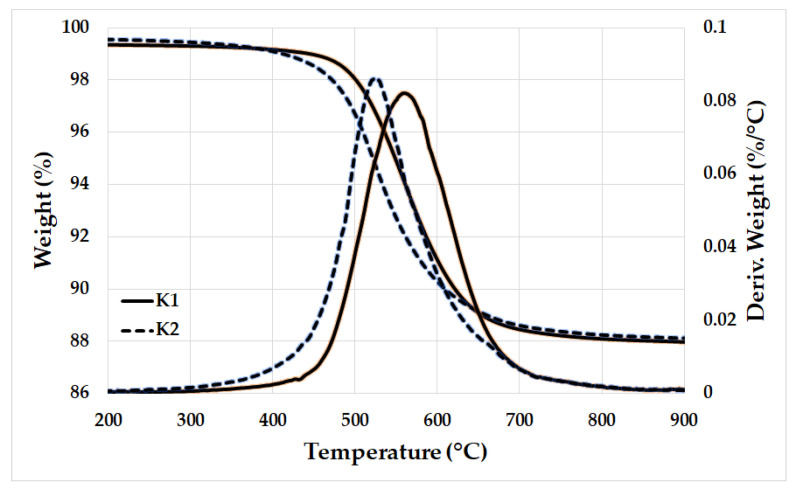
The thermal analysis curves of the K1 and K2.

**Figure 3:**
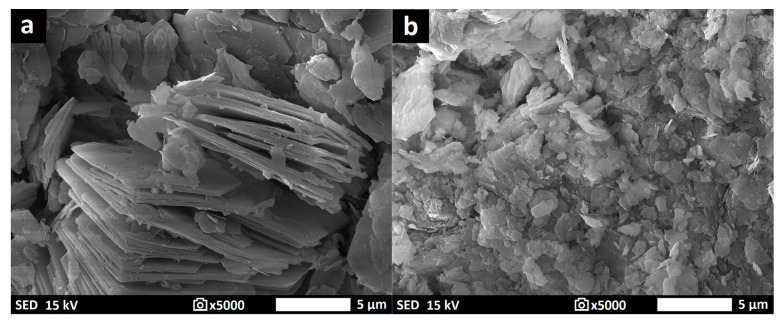
The morphology of K1 (**a**) and K2 (**b**).

Aluminosilicate raw materials, i.e., commercial purified granulated kaolin KDG (K1) and natural kaolinitic claystone W Supra (K2) obtained from Kaolin Hlubany, a.s. and České lupkové závody, a.s., in the Czech Republic, respectively. Potassium hydroxide (Lach-Ner, s.r.o., Neratovice, Czech Republic) and potassium silicate (Vodní sklo, a.s., Prague, Czech Republic) were applied as the activator. The filler (Quartz sand with grain size of 0—2 mm) was bought from Provodínské písky, a.s., in Provodín, Czech Republic. Table 1 and Table 2 show the chemical compositions and physical properties of the applied raw materials. Moreover, the thermal analysis curves, X-ray diffraction (XRD) patterns, and morphology of the aluminosilicate raw materials are displayed in Figure 1, Figure 2 and Figure 3.

### 2.2. Preparation of Aluminosilicates with Different Particle Size Distribution

**Figure 4:**
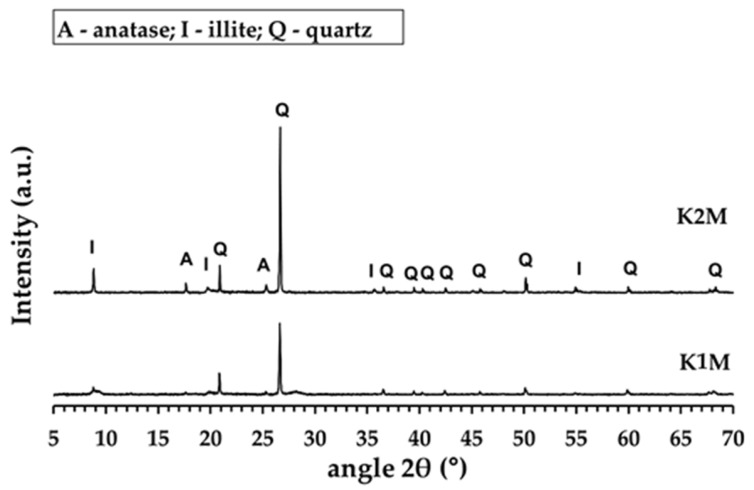
XRD patterns of calcined and ground aluminosilicate materials.

**Figure 5:**
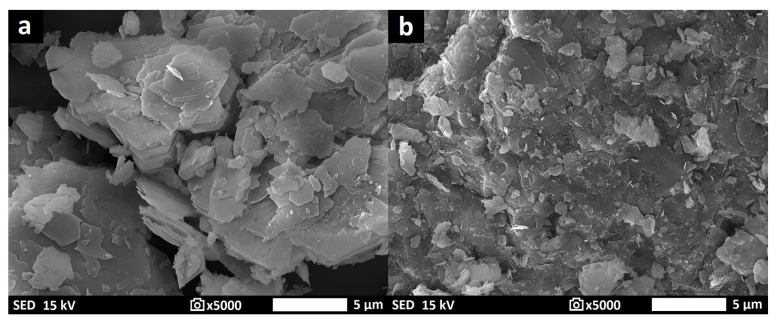
The morphology of K1M (**a**) and K2M (**b**).

Two aluminosilicate raw materials were subjected to calcination in an electric furnace (Clasic, type 5013V, Řevnice, Czech Republic) at a temperature of 750 °C for 15 min, with a heating rate of 10 °C/min. The selection of calcination conditions was based on the thermogravimetric analysis results of the aluminosilicate raw materials (Figure 2). The calcined aluminosilicate raw materials were then milled in a ball mill and subsequently sieved using a 1 mm mesh size. The resulting aluminosilicate materials were designated as K1M and K2M. Table 3 displays the chemical compositions of the obtained aluminosilicate materials. The XRD patterns and morphology of the materials are depicted in Figure 4 and Figure 5, respectively.

The pre-ground calcinates underwent additional milling using various conditions in a jet mill equipped with an air classifier (Hosokawa Alpine, Augsburg, Germany). The milling conditions were altered by adjusting the speed of the classifier to 2200, 6000, 10,000, and 20,000 rpm, resulting in four distinct fractions. One fraction was obtained solely by classifying the original pre-ground aluminosilicates into fine and coarse fractions using an air classifier, without involving the jet mill. In this case, the speed of the classifier was set at 10,000 rpm, and the air flow was maintained at 60 m^3^/h. Only the coarse fraction was utilized in the experiment. Each pre-ground aluminosilicate (K1M and K2M) yielded a total of five fractions with varying particle size distributions. The milled and classified aluminosilicates with different particle size distributions were labeled according to the classifier rotation speed as KXM-2200, KXM-6000, KXM-10000, KXM-20000, and KXM-coarse, where X represents the specific aluminosilicate materials employed. Additionally, the pre-ground aluminosilicates K1M and K2M were included as one of the fractions and further denoted as K1M-prime and K2M-prime.

### 2.3. Preparation of Geopolymers

To prepare geopolymers, the alkali activator was prepared by mixing a commercially available potassium silicate solution with solid potassium hydroxide. To eliminate any moisture absorbed during milling and storage, all fractions of the two aluminosilicates, K1M and K2M, underwent drying at 110 °C for 24 h. For producing the geopolymer binders, one fraction of aluminosilicate material was mixed with the alkali activator in a planetary mixer for 10 min. Distilled water was then added, and the mixing continued for an additional 5 min, resulting in a total water content of 30% in the geopolymer binder. For all tested aluminosilicate fractions, the weight ratio of the aluminosilicate component to the alkali activator was consistently maintained at 45:55. Immediately after mixing, the dynamic viscosity and setting time of the geopolymer binders were measured. The freshly mixed and homogeneous geopolymer binders were poured into silicon molds, vibrated to remove air bubbles, and then sealed in polyethylene bags. They were cured at 60 °C for 4 h in an electric oven. Afterward, the geopolymer binder samples were cured by molding at room temperature (20 °C) for a period of 7 days.

The geopolymer binders that were prepared had a molar ratio of Me:Al equal to 1, where Me represents an alkali metal. For series K1M, the Si:Al molar ratio was 1.7, and for series K2M, it was 1.5. The selection of the geopolymer binder composition was based on previous studies [41,47]. The geopolymer binders were identified as GB-X-Y (X and Y represented the applied aluminosilicate materials and the speed of rotation of the classifier, respectively).

To create the geopolymer composites, the geopolymer binder was mixed with quartz sand (50:50 weight ratio) for an additional 5 min. The curing conditions of the composites were the same as those for the binders. The geopolymer composites were labeled as GC-X-Y, following the same naming convention as the geopolymer binders.

### 2.4. Analytical and Testing Methods

To chemically analyze the aluminosilicate materials, a BRUKER S8 Tiger instrument with X-ray fluorescence spectrometer (XRF) capability from Bruker in Billerica, Boston, MA, USA was utilized.

XRD analysis was carried out to assess the phase composition of the aluminosilicate materials. XRD patterns were obtained using a BRUKER D8 Advanced system equipped with a BRUKER SSD 160 detector. The XRD system operated with Cu-Kα radiation, and the X-ray source was set at 40 kV and 25 mA. Scanning in the XRD analysis was conducted at a step size of 0.02°, covering an angular range from 5° to 70°, with a dwell time of 1 s.

The particle size and specific gravity of the aluminosilicate materials was determined using a laser diffractometer Mastersizer 3000 (MALVERN Instruments, Malvern, UK) and Pycnomatic ATC Evo (Microtrac, Osaka, Japan).

The BET surface area of the aluminosilicate materials was calculated using an Autosorb iQ (Quantochrome Instruments, Boynton Beach, FL, USA) by nitrogen adsorption/desorption.

To determine the pore size distributions, an AutoPore IV 9510 mercury intrusion porosimeter (Micromeritics, Unterschleißheim, Germany) was employed at 0.01 MPa to 414 MPa.

The K/Na ratio and micro-element content in the potassium silicate were analyzed using an OPTIMA 8000 provided by Perkin Elmer (Waltham, MA, USA). Conventional acid-base titration methods were applied for determination of the total amounts of Na, K, and SiO2 in the sodium silicate.

Thermal analysis of the samples, including thermogravimetry (TG) and differential thermal analysis (DTA), was carried out by a Discovery Series thermal analysis system (TA Instruments) in nitrogen atmosphere at a flow rate of 20 mL/min and a heating rate of 10 °C/min up to 900 °C.

To assess the leaching of aluminosilicate materials in the alkaline activator, plastic beakers (TPX) were employed. The aluminosilicate fractions were mixed with the alkaline activator at a weight ratio of 45:55. The mixing process was conducted at room temperature for 15 min, utilizing an Ika EUROSTAR digital stirrer shaft (IKA in Staufen, Germany). Following mixing, the samples were centrifuged, leading to the separation of the liquid (supernatant) and solid (sediment) phases. The supernatants were subjected to aluminum concentration analysis using ICP-OES.

A rotary rheometer (Rheotest RN 4.1, Rheotest Medingen, Ottendorf-Okrilla, Germany) was applied for determination of the dynamic viscosities of the geopolymer binders applying a 38 mm diameter cylinder at 25 °C and a shear rate of 300 s^−1^ for 600 s.

The initial, final, and setting times of the geopolymer binders were determined using the Automatic apparatus Vicatronic (MATEST, Treviolo, Italy) in accordance with the EN 480-2 standard.

To study the surface morphology of the materials, scanning electron microscope (SEM) analysis was carried out using JSM-IT500HR (JEOL in Tokyo, Japan).

For the determination of mechanical properties, LabTest 6.200 (Labortech in Opava, Czech Republic) was employed. Flexural strength was assessed through a three-point bending test on six geopolymer composite samples (20 mm × 20 mm × 160 mm), with a crosshead speed of 0.25 mm/min. Compressive strength and modulus of elasticity on six prismatic geopolymer composite samples (30 mm × 30 mm × 64 mm) were measured following the ISO 1920-10 standard at a loading speed of 0.25 mm/min.

## 3. Results and Discussion

### 3.1. Properties of Calcined and Milled Aluminosilicates

Figure 4 displays the XRD patterns of the calcined K1M and K2M samples. The XRD results indicate that the crystalline kaolinite in both the K1M and K2M samples transformed completely into amorphous metakaolinite during the calcination process. Additionally, both samples contained crystalline impurities such as quartz, anatase, and illite.

The SEM results of the K1M and K2M samples are shown in Figure 5. The particle morphology of the K1M sample differed from that of the K2M sample. The K1M sample had a higher proportion of particles with a high aspect ratio compared to the K2M sample. This variation might be attributed to the different particle morphology of the initial materials. During grinding, smaller and less structured plates were separated with more difficulty than larger and more structured ones.

**Figure 6:**
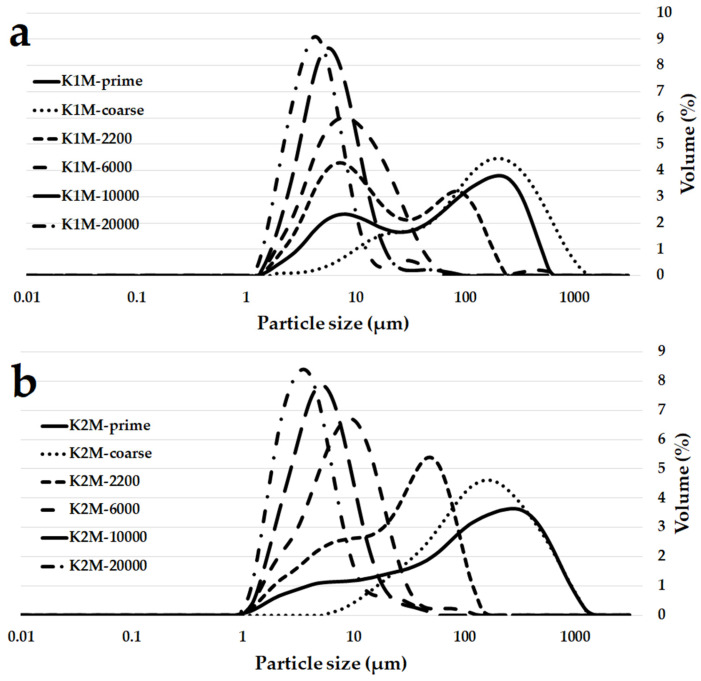
Particle size distributions of the individual fractions of K1M (**a**) and K2M (**b**).

Figure 6 demonstrates the results of the particle size distribution analysis for K1M and K2M samples, conducted using laser diffraction. The milled materials exhibited particle size distributions different from those of the initial raw materials. However, the particle size distributions of the individual fractions were similar between the K1M and K2M samples. Notably, the particle size distributions of the K1M-10000 and K2M-10000 samples closely resembled those of high-quality commercially available metakaolinite sources (Mefisto K05 and Mefisto L05), which were previously tested and industrially produced from similar raw materials [41].

Table 4 provides the physical properties of the calcined and milled aluminosilicate samples. Calcination and milling processes induced changes in specific gravity. Four factors, namely dehydroxylation, impurity oxidation, shrinkage, or expansion, could be influenced by the alteration in specific gravity [54]. As expected, the bulk density significantly decreased with decreasing grain size. Moreover, the K2M samples exhibited noticeably higher bulk densities compared to the K1M samples, which can be due to the aforementioned differences in particle morphology.

### 3.2. Leachability Test

**Figure 7:**
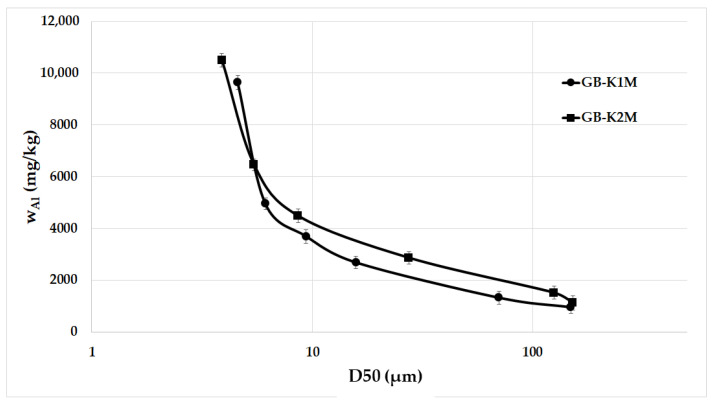
Al concentrations in supernatants after leaching milled aluminosilicates K1M and K2M with different particle sizes in an alkaline activator.

Figure 7 illustrates the concentrations of aluminum (Al) in the liquid portion after treating milled aluminosilicates (K1M and K2M) composed of different particle sizes with an alkaline activator. The analysis focused on Al concentrations because detecting small changes in silicon (Si) concentrations during leaching was unreliable due to the high Si concentration in the alkaline activator. The analysis results were adjusted using the blank value. The findings indicate that as the average particle size decreases, the leaching rate significantly increases. The samples with the smallest mean particle size exhibited the highest Al concentration in the liquid portion. This enhanced leachability can be attributed to the larger surface area of finer particles compared to coarser ones (Table 4). Kuenzel et al. [40] conducted a study comparing the leachability of commercially available metakaolins with different particle size distributions and found that metakaolin with larger particles exhibited the least leachability. In our study, the highest concentrations of Al (9640 mg/kg and 10,490 mg/kg) in the liquid portion were observed in samples K1M-20000 and K2M-20000, which had mean particle sizes of 4.6 µm and 3.9 µm, respectively. These elevated Al concentration values could be due to the highly reactive Al and Si present in metakaolin, which is commonly used in the preparation of geopolymers. The relationship between Al concentration in the liquid portion and aluminosilicate particle size was nearly identical for both examined aluminosilicate raw materials.

### 3.3. Rheological Properties

**Figure 8:**
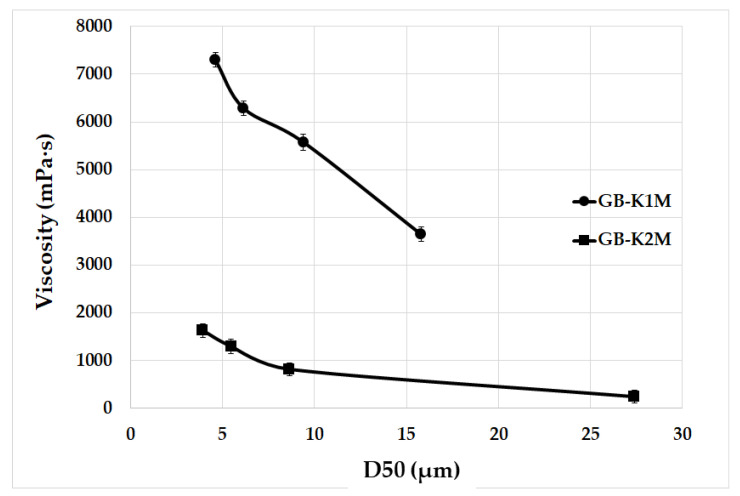
Viscosities of geopolymer binders prepared from milled aluminosilicates K1M and K2M with different particle sizes depending on the average particle size.

The rotary rheometer was used to investigate the dynamic viscosity of geopolymer binder samples immediately after their preparation. Figure 8 displays the results of investigating how different particle size distributions of aluminosilicates affect the dynamic viscosity of the geopolymer binder samples. However, dynamic viscosity measurements were not conducted for samples GB-K1M-prime, GB-K2M-prime, GB-K1M-coarse, and GB-K2M-coarse due to the presence of excessively large particles that could potentially damage the rheometer’s rotating cylinder. In both tested series, the dynamic viscosity of the geopolymer binders decreased as the mean particle size increased. For the GB-K1M samples, the dynamic viscosity decreased from 7302 mPa·s to 3652 mPa·s as the mean particle size increased, while for the GB-K2M samples, it decreased from 1631 mPa·s to 246 mPa·s. Changes in dynamic viscosity can be attributed to the bulk density of the tested materials, as well as the size and shape of the particles. Finer-grained samples have a larger surface area per unit mass compared to coarser-grained samples. The decrease in dynamic viscosity with increasing mean particle size is in agreement with the findings of Boháč et al. [55], who observed similar results in Portland cement pastes with varying particle size fractions of metakaolin. Geopolymer binders prepared from the GB-K2M series exhibited significantly lower dynamic viscosity (up to 4.5 times lower in the case of samples with the finest grain size) compared to those prepared from the GB-K1M series. This difference can be attributed to the distinct particle morphology (Figure 5). As mentioned earlier, the particles in the K1M sample had a higher aspect ratio compared to those in the K2M sample. This is due to a more pronounced arrangement of kaolinite plates in kaolin, which readily break up into individual plates during milling [41,56].

### 3.4. Determination of Setting Time

Table 5 presents the initial, final, and actual setting times of the geopolymer binders produced from milled aluminosilicates K1M and K2M with varying particle sizes at 25 °C. The particle size had a notable impact on the hardening process of the geopolymer binders. As the particle size decreased (finer particles), the initial setting time for hardening significantly decreased as well (120 min for the GB-K1M series and 70 min for the GB-K2M series). The actual setting time was also slightly affected, with values of 25 min for the GB-K1M series and 20 min for the GB-K2M series. The enhanced leachability of finer-grained samples likely leads to a faster geopolymerization process, resulting in an earlier onset of hardening [21]. Additionally, the type of aluminosilicate used also influences the setting of the geopolymers. Geopolymer binders prepared from the calcined kaolin series (K1M) exhibited a hardening onset that was up to twice as slow as that of binders prepared from the calcined kaolinitic clay series (K2M) at 25 °C. These significant changes are likely attributed to the above-mentioned particle morphology of individual aluminosilicate particles. Furthermore, the presence of impurities in the aluminosilicate could also impact the setting time.

### 3.5. Mechanical Properties

**Figure 9:**
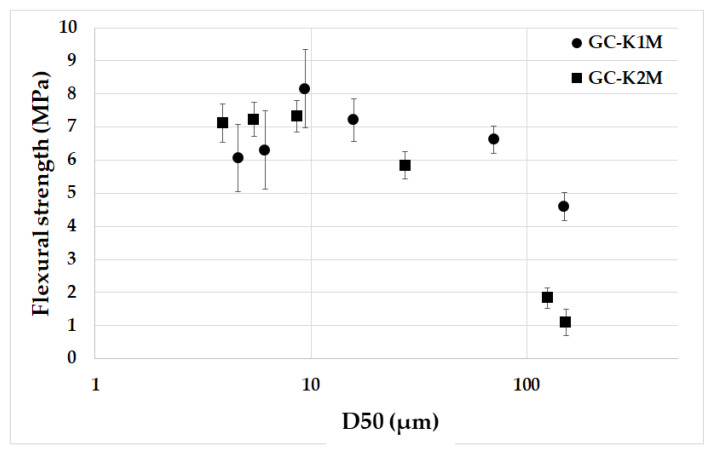
Flexural strength of the geopolymer composites prepared from milled aluminosilicates K1M and K2M with different particle sizes depending on the average particle size.

Figure 9 illustrates the relationship between the flexural strength of geopolymer composites and the average particle size of milled aluminosilicates K1M and K2M. Both series of samples exhibited a decrease in flexural strength as the mean particle size increased. This decline is likely attributed to the presence of undissolved larger platy particles of aluminosilicates, which weaken the structure of the geopolymer matrix, leading to cracks and internal damage and ultimately reducing the flexural strength [55]. Although the flexural strength of the two finest samples in the GC-K1M series (GC-K1M-10000 and GC-K1M-20000) may appear lower compared to the GC-K1M-6000 sample, statistical analysis within the standard deviation indicated no significant difference between the samples, making them comparable. The highest flexural strength in the GC-K1M series was achieved in the GC-K1M-6000 sample with a mean particle size value of 10 µm, measuring 9.14 MPa. However, the influence of particle size on flexural strength was only observable up to a mean particle size of 10 µm for the GC-K2M series. Beyond that threshold, there were no significant differences in flexural strength (around 7.2 MPa) among samples prepared from materials with a mean particle size smaller than 10 µm (GC-K2M-6000, GC-K2M-10000, and GC-K2M-20000).

**Figure 10:**
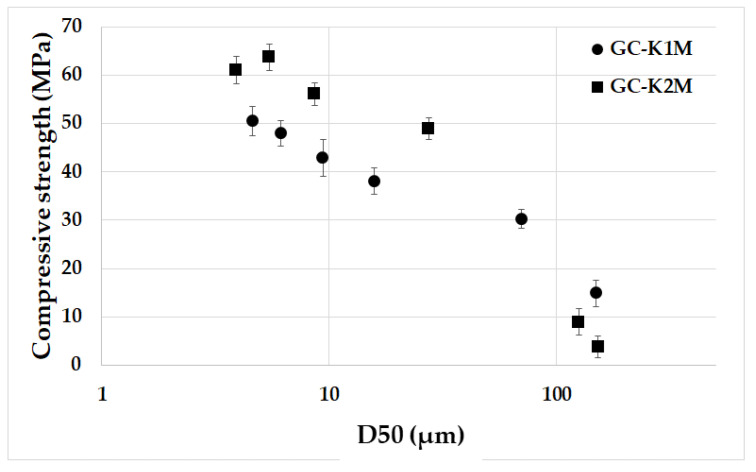
Compressive strength of the geopolymer composites prepared from milled aluminosilicates K1M and K2M with different particle sizes depending on the average particle size.

**Figure 11:**
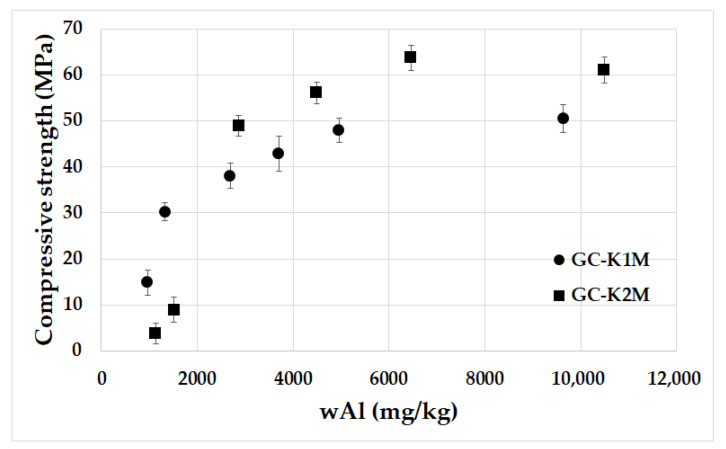
Compressive strength of the geopolymer composites prepared from milled aluminosilicates K1M and K2M with different particle sizes depending on leaching.

Figure 10 depicts the compressive strength of geopolymer composites prepared from milled aluminosilicates K1M and K2M with varying particle sizes based on the average particle size. The results confirm previous findings, showing a significant increase in compressive strength as the mean particle size decreased for geopolymer composites derived from both types of aluminosilicates. The compressive strength rose from an average of 10 MPa to 50–60 MPa. Finer particles exhibited higher dissolution rates (Figure 7), resulting in the formation of more geopolymer gel. This contributes to a stronger structure within the geopolymer matrix and subsequently enhanced the overall compressive strength as is shown in Figure 11 [57]. Xu and Van Deventer [39] have previously confirmed that if aluminosilicates show a higher extent of dissolution, then the geopolymers demonstrate better compressive strength after geopolymerization. Additionally, finer particles possess a larger specific surface area (Table 4), which potentially leads to reduced water content in the geopolymer matrix. Among the GC-K1M series samples, the highest compressive strength of 50.54 MPa was observed in the GC-K1M-20000 sample with a mean particle size of 4.6 µm. Similar to the flexural strength, no significant differences were noted in the compressive strength of samples with an average particle size below 10 µm for the GC-K2M series. The GC-K2M-10000 sample exhibited the highest compressive strength value (63.7 MPa) within the GC-K2M series, with an average particle size of 5.5 µm. The compressive strengths of GC-K2M-6000 and GC-K2M-20000 samples were 56.10 MPa and 60.1 MPa, respectively. It is clear that geopolymer composites lacking small aluminosilicate particles display poor mechanical properties. Furthermore, the samples prepared from calcined kaolinitic claystone (K2M series) exhibited higher compressive strengths compared to those prepared from calcined kaolin (K1M series), which is in agreement with previous research [41,56]. The measured compressive strength values are consistent with published results for geopolymer composites of similar compositions (55–65 MPa) [48,58,59,60].

**Figure 12:**
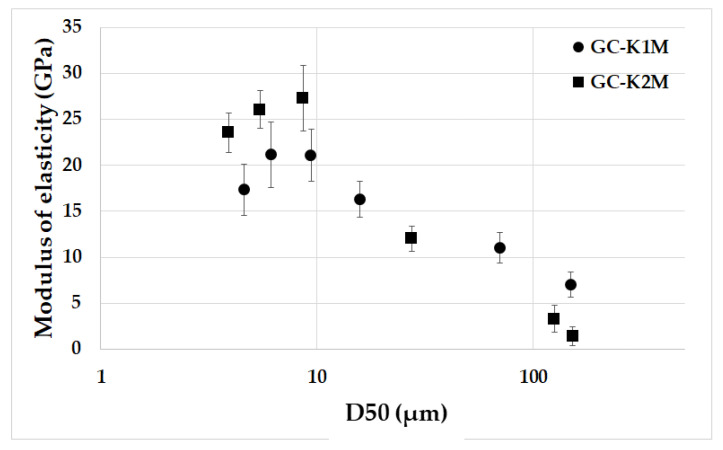
Moduli of elasticity of the geopolymer composites prepared from milled aluminosilicates K1M and K2M with different particle sizes depending on the average particle size.

The results for the elastic moduli of geopolymer composites prepared from milled aluminosilicates K1M and K2M with varying particle sizes based on the average particle size are presented in Figure 12. The relationship between elastic moduli and mean particle size observed in the geopolymer composites was similar to that of compressive strength. This implies that the elastic modulus increased as the mean particle size decreased, and there were no significant differences among samples prepared from materials with a mean particle size below 10 µm. The samples with the smallest average particle size from both series (GC-K1M-20000 and GC-K2M-20000) displayed a slight decrease in the elastic modulus compared to samples GC-K1M(K2M)-10000, but these results fall within the range of error indicated by the standard deviation. At room temperature, the geopolymer composites GC-K1M-6000, GC-K1M-10000, GC-K2M-6000, and GC-K2M-10000 exhibited elastic moduli of 21.08 GPa, 21.16 GPa, 27.3 GPa, and 26.06 GPa, respectively. These measured values align with published results for elastic moduli of geopolymer composites with similar compositions (20–25 GPa) [59,60].

### 3.6. Porosity

**Figure 13:**
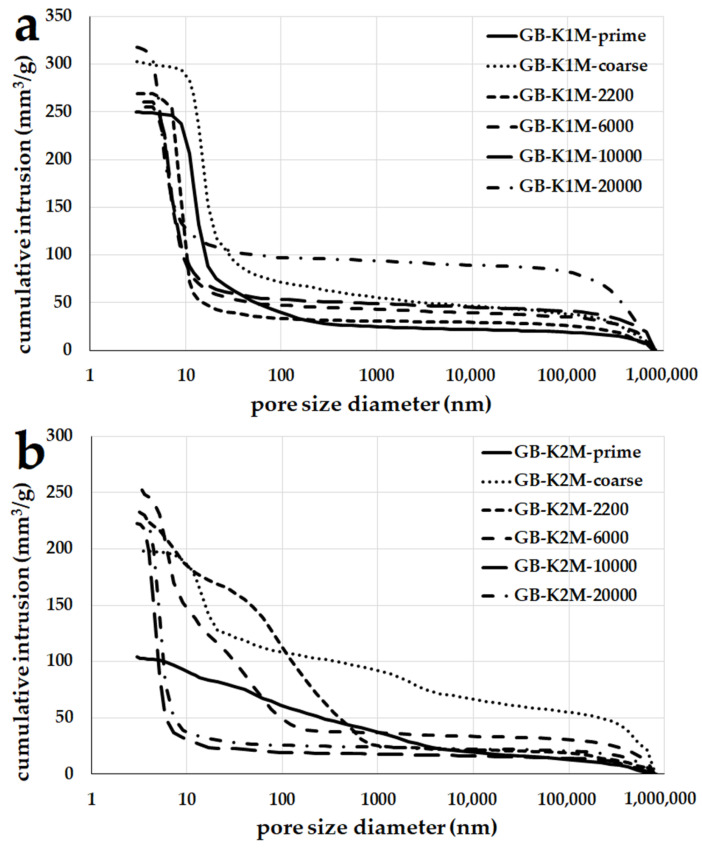
Mercury intrusion porosimetry of the geopolymer binders prepared from milled aluminosilicates K1M (**a**) and K2M (**b**) with different particle sizes.

Figure 13 depicts the cumulative intrusion volume of mercury during testing, applied to geopolymer binders produced from milled aluminosilicate materials K1M (Figure 13a) and K2M (Figure 13b). In geopolymer binders derived from K1M at ambient laboratory temperature, the predominant presence of mesopores up to 10 nm was observed. No significant variations were noted among individual samples within the K1M series. On the other hand, in geopolymer binders prepared from K2M, it is evident that samples with larger grain sizes also exhibited larger pores ranging from 100 to 1000 nm. Only samples GB-K2M-10000 and GB-K2M-20000 primarily contained mesopores up to 10 nm.

**Figure 14:**
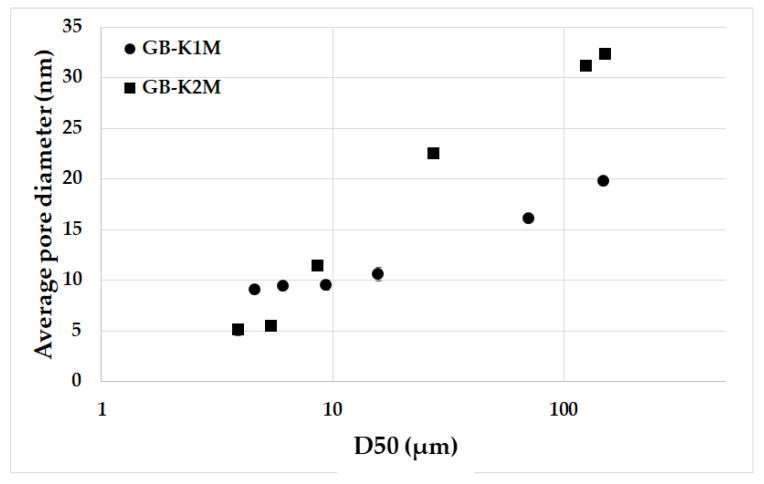
Average pore diameter of geopolymer binders prepared from milled aluminosilicates K1M and K2M with different particle sizes depending on the average particle size.

Figure 14 displays the average pore diameter of hardened geopolymer binders produced from milled aluminosilicates K1M and K2M with different particle sizes based on the average particle size. It was observed that there was a decrease in average pore diameter as the average particle size decreased for both sample series. The average pore diameter reduced from 19.8 nm (GB-K1M-coarse) and 32.3 nm (GB-K2M-coarse) to 9.1 nm (GB-K1M-20000) and 5.1 nm (GB-K2M-20000). In samples consisting of coarser aluminosilicate fractions, leachability was significantly lower (Figure 7), resulting in only a portion of the porous aluminosilicate particles dissolving during leaching [21]. The remaining undissolved residues gradually became filled with a geopolymer matrix [48]. The changes in average pore diameter can be attributed to the availability of more large pores to be filled with the geopolymer matrix.

### 3.7. Morphology

**Figure 15:**
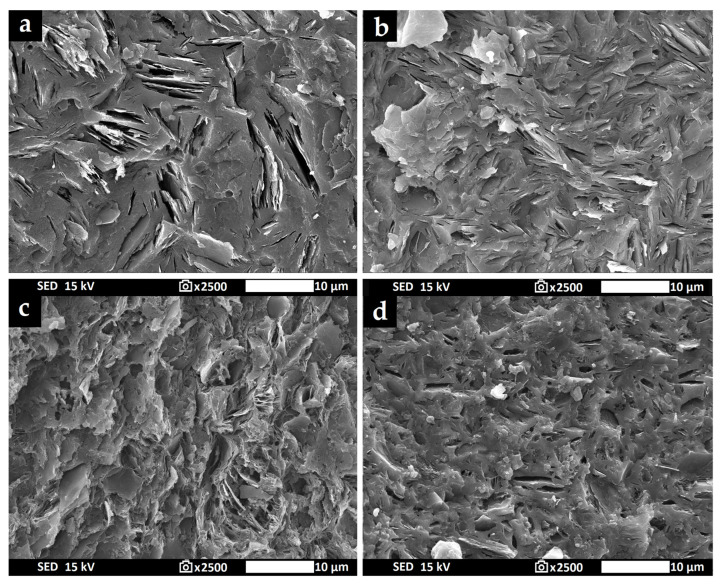
Micrographs of geopolymer binders prepared from milled aluminosilicates K1M and K2M (**a**)—GB-K1M-prime; (**b**)—GB-K1M-20000; (**c**)—GB-K2M-prime; (**d**)—GB-K2M-20000.

Figure 15 shows the surface characteristics of geopolymer binders prepared from milled aluminosilicates K1M and K2M. Only two samples are displayed from each series of aluminosilicates tested: geopolymer binders derived from the prime material and from the material milled at 20,000 rpm (Figure 15a—GB-K1M-prime; Figure 15b—GB-K1M-20000; Figure 15c—GB-K2M-prime; Figure 15d—GB-K2M-20000). No notable distinctions were observed between the two samples within each aluminosilicate series. As the remaining samples of geopolymer binders possessed a very similar structure, only these two samples are presented. In this report, all displayed samples revealed a compact, heterogeneous, amorphous, and geopolymer matrix containing numerous undissolved metakaolinite plates. The geopolymer matrices did not feature a significant number of visible pores. No substantial differences were identified between samples utilizing different types of aluminosilicates.

## 4. Conclusions

The present research investigated the effect of different particle size distributions of aluminosilicates on the mechanical and microstructural properties of metakaolinite-based geopolymer composites and binders. Based on the obtained results, the following conclusions can be drawn:

- The Al leaching rate increased significantly as the average particle size decreased, leading to a decrease in bulk density;
- The dynamic viscosity of fresh geopolymer binders decreased as the mean particle size increased. Geopolymer binders prepared from calcined kaolin had much lower dynamic viscosity compared to binders prepared from calcined kaolinitic claystones;
- Smaller particle sizes resulted in a shorter initial setting time for the hardening process of geopolymer binders;
- The influence of particle size on the mechanical properties of geopolymer composites was observed up to a mean particle size of 10 µm. Beyond that size, mechanical properties decreased as the mean particle size increased;
- Geopolymer binders prepared from calcined kaolin had mesopores up to 10 nm, while binders from calcined kaolinitic claystone with larger particle sizes contained pores ranging from 100 to 1000 nm. Average pore diameter decreased as the average particle size decreased;
- The studied geopolymer binders, with different particle size distributions, showed no significant differences in their morphological structure.

Overall, the results clearly indicate that geopolymer binders prepared from aluminosilicates with finer particle fractions (mean particle size ranging from 3.9 to 10 µm) displayed superior properties compared to those prepared from aluminosilicates with coarser particle fractions. These advantages include faster hardening, smaller average pore diameter, and higher mechanical properties.

## Figures and Tables

**Table 1:** Chemical composition (wt.%) of used raw materials.

Material	Material Composition (%)													
	^a^ LOI	H_2_O	Al_2_O_3_	SiO_2_	Fe_2_O_3_	CaO	MgO	Na_2_O	K_2_O	TiO_2_	P_2_O_5_	ZrO_2_	SO_3_	Cr_2_O_3_
K1	12.28	-	34.7	50.7	0.58	0.13	0.45	-	0.74	0.37	0.01	0.01	0.04	0.01
K2	11.88	-	38.1	46.6	0.67	0.16	0.13	-	0.75	1.31	0.05	0.03	0.28	0.02
Potassium silicate	-	62.18	0.04	25.2	0.75	-	-	0.25	12.4	-	-	-	-	-

^a^ LOI = Loss on ignition.

**Table 2:** Physical properties of the raw materials (K1 and K2).

Material	Specific Gravity	BET Surface Area	Pore Volume	Average Pore Size
		(m^2^/g)	V (mm^3^/g)	R (nm)
K1	2630	14.8	314	106.6
K2	2 640	11.1	58	19.7

**Table 3:** Chemical composition (wt.%) of calcined and ground aluminosilicate materials.

Material	^a^ LOI	Al_2_O_3_	SiO_2_	Fe_2_O_3_	CaO	MgO	ZnO	K_2_O	TiO_2_	P_2_O_5_	ZrO_2_	SO_3_	Cr_2_O_3_
K1M	0.35	38.8	58.1	0.74	0.17	0.52	0.01	0.84	0.48	0.01	0.01	0.04	0.01
K2M	0.35	41.8	53.9	0.86	0.17	0.11	0.01	0.89	1.55	0.06	0.03	0.24	0.03

^a^ LOI = Loss on ignition.

**Table 4:** Physical properties of calcined, milled, and classified aluminosilicates.

Material	Specific Gravity	BET Surface Area	Bulk Density	Particle Size		
		(m^2^/g)	(kg/m^3^)	D10 (µm)	D50 (µm)	D90 (µm)
K1M-coarse	2650	11.6	741	18.3	149	523
K1M-prime	2660	12.1	557	5.64	70.3	318
K1M-2200	2810	12.5	393	4.14	15.8	111
K1M-6000	2710	12.8	277	3.49	9.40	27.4
K1M-10000	2610	13.1	246	2.92	6.14	13.2
K1M-20000	2670	13.3	219	2.30	4.60	9.74
K2M-coarse	2620	10.3	1276	31.2	152	551
K2M-prime	2610	10.8	1170	6.66	125	221
K2M-2200	2850	11.6	690	3.97	27.4	79.3
K2M-6000	2810	12.0	488	2.88	8.62	21.2
K2M-10000	2990	13.7	400	2.40	5.45	12.6
K2M-20000	2950	14.7	326	1.93	3.90	9.29

**Table 5:** The initial, final, and real setting time of geopolymer binders produced from milled aluminosilicates K1M and K2M with different particle sizes at 25 °C and 95% humidity.

Measurement Conditions	Sample	Setting Time (min)		
		Intial	Final	Real
25 °C, 95% humidity	GB-K1M-coarse	470	640	170
	GB-K1M- prime	507	572	65
	GB-K1M-2200	491	526	35
	GB-K1M-6000	464	504	40
	GB-K1M-10000	404	454	50
	GB-K1M-20000	390	436	46
	GB-K2M-coarse	290	320	30
	GB-K2M-prime	290	315	25
	GB-K2M-2200	330	420	90
	GB-K2M-6000	325	375	50
	GB-K2M-10000	222	266	44
	GB-K2M-20000	220	255	35

## Data Availability

The data presented in this study are available on request from the corresponding author.
